# Berberine promotes XIAP-mediated cells apoptosis by upregulation of miR-24-3p in acute lymphoblastic leukemia

**DOI:** 10.18632/aging.102813

**Published:** 2020-02-13

**Authors:** Jian Liu, Zhiwei Chen, Yunping Cui, Huixia Wei, Zhenjing Zhu, Fengxia Mao, Yingchao Wang, Yufeng Liu

**Affiliations:** 1 Department of Pediatrics, The First Affiliated Hospital of Zhengzhou University, Zhengzhou 450052, People’s Republic of China

**Keywords:** Berberine, acute lymphoblastic leukemia, X-linked inhibitor of apoptosis protein, MiR-24-3p, PIM-2

## Abstract

Background: Berberine (BBR) has gained considerable attention because of its anti-tumor activity. BBR can induce apoptosis of acute lymphoblastic leukemia (ALL) cells through the MDM2/p53 pathway. However, the effects of BBR on those ALL patients with p53 deficiency remain unclear.

Results: We found that BBR reduced ALL cell viability and induced apoptosis in p53-null EU-4 and p53-mutant EU-6 cells by downregulating X-linked inhibitor of apoptosis protein (XIAP), which is increased in ALL tissues and cells. BBR-induced cell apoptosis was attenuated by inhibition of XIAP that was controlled by PIM-2. Mechanistic studies showed that BBR treatment induced an enhancement of miR-24-3p. PIM-2 is a direct target of miR-24-3p. Blockade of PIM-2 or miR-24-3p reversed BBR-induced cell apoptosis. *In vivo* studies, BBR remarkably alleviated leukemia conditions in a EU4 xenograft mouse model, whereas inhibition of miR-24-3p significantly reversed the effects of BBR in the leukemia condition.

Conclusions: miR-24-3p/PIM-2/XIAP signaling contributes to BBR-mediated leukemia mitigation in p53-defect ALL, which should be further developed as a treatment strategy in ALL patients with p53 deficiency.

Methods: Cell viability and apoptosis were determined using CCK-8 and TUNEL assays, respectively. The dual-luciferase reporter gene system was used to determine the interaction between miR-24-3p and 3′-untranslated regions (UTRs) of PIM-2.

## INTRODUCTION

Berberine (BBR, C20H19NO5, a 5, 6-dihydro-dibenzo[a,g]quinolizinium derivative) is a small molecule alkaloid purified from Chinese herbs. For decades, BBR has been proven to have anti-inflammatory and anti-tumor effects in a broad range of human cancers [1]. Numerous research results have demonstrated the beneficial effects of BBR on cellular metabolism through multiple mechanisms. For example, BBR could relieve glomerular podocyte injury by inhibiting mitochondrial fission and dysfunction in diabetic kidney disease [2]. BBR attenuates the development of diet-induced obesity via up-regulating fibroblast growth factor 21, which is an important metabolic regulator [3]. BBR possesses the ability to inhibit human cancers through various mechanisms of action. It has been demonstrated that BBR inhibits proliferation and induces apoptosis of cancer cells, such as nasopharyngeal carcinoma, gastric cancer, hepatocellular carcinoma, and colorectal adenocarcinoma [4–7]. BBR induces apoptosis in acute lymphoblastic leukemia (ALL) cells that are positive for wild-type p53 by downregulating the MDM2 oncoprotein, which negatively regulates p53 [8]. Further study indicated that BBR induces p53-independent apoptosis in p53-null leukemia cells by inhibiting X-linked inhibitor of apoptosis protein (XIAP) [9].

XIAP, belonging to the inhibitor of apoptosis protein (IAP) family, plays an important role in oncogenesis by protecting cancer cells from apoptosis via inhibition of the activated forms of caspase-3, -7, and -9. Downregulation of XIAP increases the apoptosis of cancer cells induced by multi-factor stimulation, including tumor necrosis factor-related apoptosis-inducing ligand (TRAIL) and anti-tumor drugs [10]. High expression of XIAP has been found to be related to chemotherapy resistance of ALL, and inhibition of XIAP improves the sensitivity of ALL cells to prednisone in vitro [11]. Currently, available evidence demonstrates that XIAP inhibitors effectively promote apoptosis in ALL cells, supporting their potential as therapeutic agents for ALL treatment. The mechanism(s) by which BBR induces p53-independent, XIAP-mediated apoptosis in leukemia cells remains unclear.

PIM-2 kinase expression was found to be significantly increased in bone marrow samples of ALL patients [12]. Also, PIM-2 was reported to reduce cell apoptosis in ALL cells. It showed that inhibition of PIM-2 kinase activity further promotes ALL cell apoptosis induced by metformin [13]. A recent study suggested that PIM-2 cooperates with downstream factor XIAP, and thus inhibits the apoptosis of prostate cancer cells [14]. And this raises the possibility that PIM-2/XIAP axis plays a role in BBR-induced apoptosis of ALL cells.

MicroRNAs (miRNAs), which are a kind of small non-coding RNA molecules (21–23 nucleotides), have been widely investigated for their role in physiological and pathological processes, including development, differentiation, proliferation, apoptosis, secretion, and metabolism. Although they are not translated, mature miRNAs typically regulate biological processes by inhibiting their target mRNA by interacting with the 3′-untranslated regions (UTRs) of target mRNA. MiRNAs such as miR-101, miR-122, miR-21-3p, and miR-106b/25 cluster also play a critical role in anti-tumor mechanism of BBR, [15–18]. However, whether BBR-induced inhibition of PIM-2/XIAP axis is related to miRNAs in ALL cell apoptosis, is poorly understood.

In this study, we examined the effects of BBR on apoptosis of p53-null leukemia cells EU-4 and p53-mutant leukemia cells EU-6, and investigated the mechanism of BBR inducing XIAP-mediated apoptosis. The data suggested that BBR inhibited PIM-2 expression by upregulating miR-24-3p, which in turn downregulated XIAP and induced apoptosis in EU-4 and EU-6 cells.

## RESULTS

### BBR inhibits survival and induces apoptosis in ALL cells

**Figure 1:**
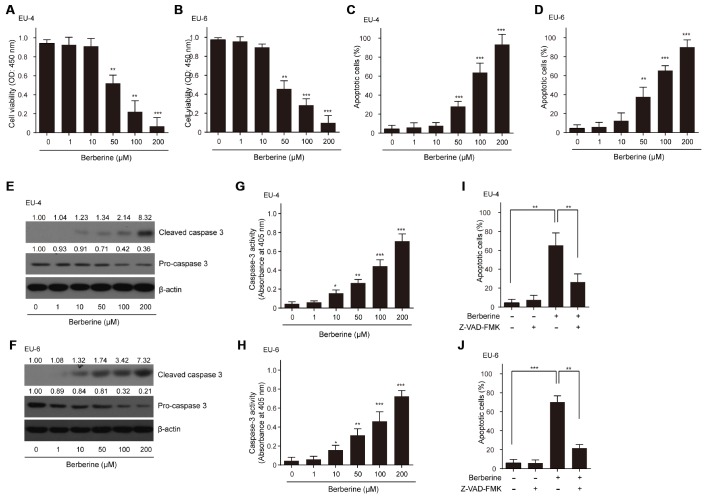
**Effects of BBR on the survival and apoptosis of ALL cells.** ALL EU-4 and EU-6 cells were treated with BBR (0, 1, 10, 50, 100, 200 μM) for 24 h. (**A**–**B**) Cell viability was analyzed by the CCK-8 assay. (**C**–**D**) Cell apoptosis was detected by TUNEL assay and the percentage of apoptotic cells was summarized. (**E**–**F**) Representative protein expression of the cleaved caspase-3 and pro-caspase-3 in cells treated with BBR at the indicated concentrations. Numerical label above each protein band indicates the fold change relative to expression of caspase-3 in the cells without BBR treatment. (**G**–**H**) Caspase-3 activity in the cells with BBR at the indicated concentrations was determined using the Caspase-3 Colorimetric Assay Kit. (**I**–**J**) ALL cells EU-4 and EU-6 cells were incubated 24 h with 50 μM BBR in the presence or absence of z-VAD-FMK (50 μM). Cell apoptosis was measured by TUNEL assay. Results are expressed as mean ± SD **p<0.01, ***p<0.001, compared to control group; *p<0.05, **p<0.01, ***p<0.001, compared to indicated group.

As expected, different concentrations of BBR (0-200 μM) decreased the viability of ALL cells EU-4 and EU-6 in a dose-dependent manner (Figure 1A and 1B). Also, we found that BBR treatment significantly induced cell apoptosis of EU-4 and EU-6 (Figure 1C and 1D). Consistently, increased cleavage of caspase-3, as well as elevated caspase-3 activity was detected in cells after BBR treatment (Figure 1E–1H). To determine whether BBR-induced apoptosis was dependent on caspase activity, ALL cells EU-4 and EU-6 cells were incubated with 50 μM BBR in the presence or absence of z-VAD-FMK, a broad-range caspase inhibitor. As shown in Figure 1I and 1J, z-VAD-FMK prevented BBR-induced apoptosis in both EU-4 and EU-6 cells. These data confirmed that BBR induced apoptosis of EU-4 and EU-6 cells, at least in part, by caspase activation.

### XIAP is implicated in the BBR-induced apoptosis in ALL cells

**Figure 2:**
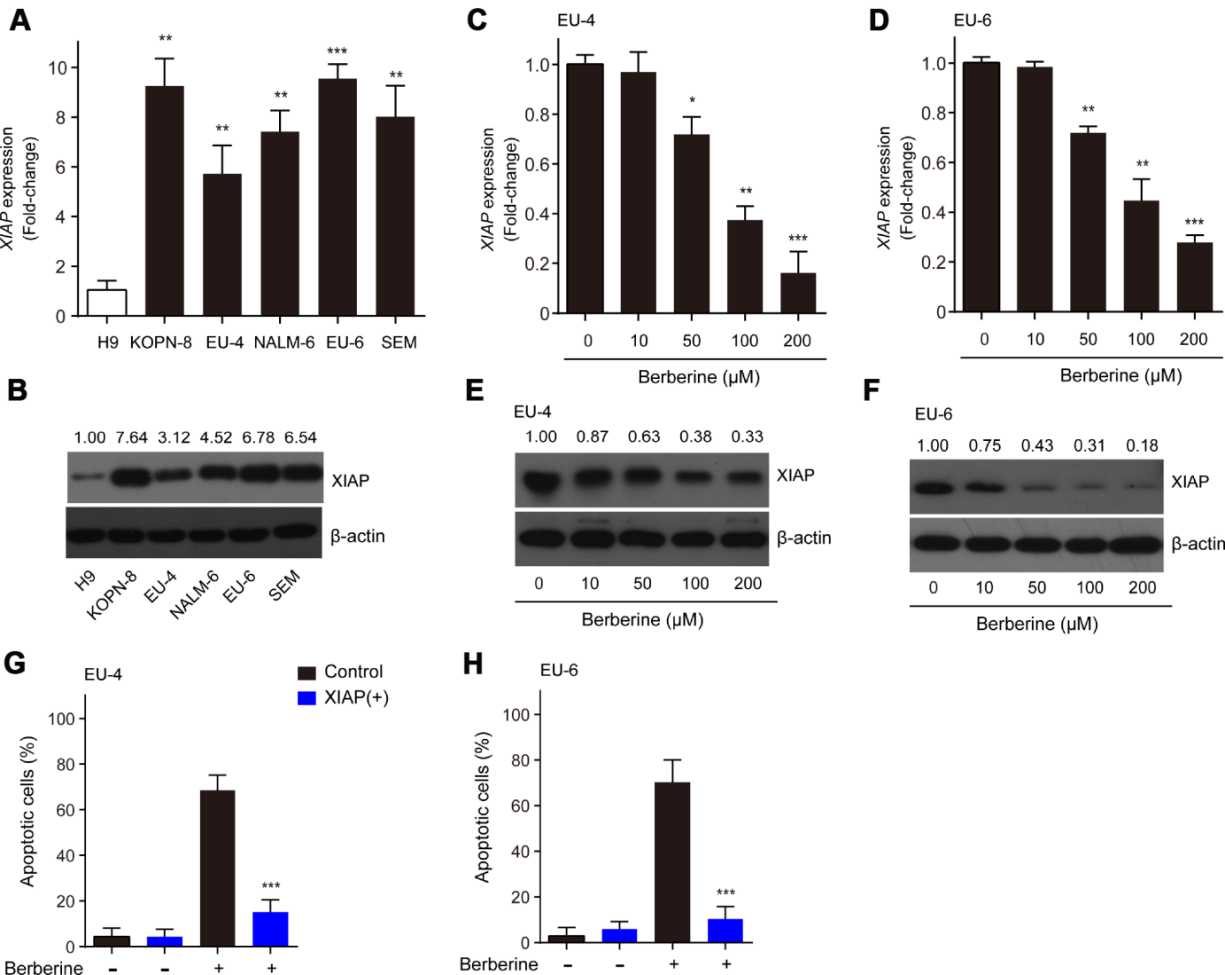
**XIAP is implicated in the BBR-induced apoptosis in ALL cells.** (**A**–**B**) Relative expression at the mRNA and protein levels of XIAP in ALL cells, KOPN-8, EU-4, NALM-6, EU-6, and SEM. H9 human lymphoma cells served as a control. EU-4 and EU-6 cells were treated by BBR at the indicated concentrations for 24 h. (**C**–**D**) Expression of XIAP mRNA in EU-4 and EU-6 cells. (**E**–**F**) Representative western blot analysis showing the effect of BBR on the expression of XIAP in EU-4 and EU-6 cells. (**G**–**H**) EU-4 and EU-6 cells were transfected with vector containing XIAP, or negative vector, and subsequently treated with 50 μM BBR for 24 h. Cell apoptosis was measured by TUNEL assay. The numbers above the western blots band are the quantification of the gray value normalized to control group. Results are expressed as mean ± SD. *p<0.05, **p<0.01, ***p<0.001, compared to the control or indicated group.

XIAP has been reported to be associated with apoptotic signaling in ALL cells by inhibiting caspase activation [19]. In the present study, we found that the relative mRNA expression and protein levels of XIAP in ALL cells, KOPN-8, EU-4, NALM-6, EU-6 and SEM were significantly higher than those in human lymphoma cells H9 (Figure 2A and 2B). Next, we investigated the expression of XIAP in BBR-treated EU-4 and EU-6 cells. In response to BBR stimulation, the relative mRNA expression and protein levels of XIAP were significantly downregulated (Figure 2C–2F). In addition, following overexpression of XIAP, the BBR-induced apoptosis of EU-4 and EU-6 cells was significantly decreased (Figure 2G and 2H). The data show that XIAP is implicated in BBR-induced ALL cells apoptosis.

### BBR induces XIAP-mediated apoptosis through PIM-2

**Figure 3:**
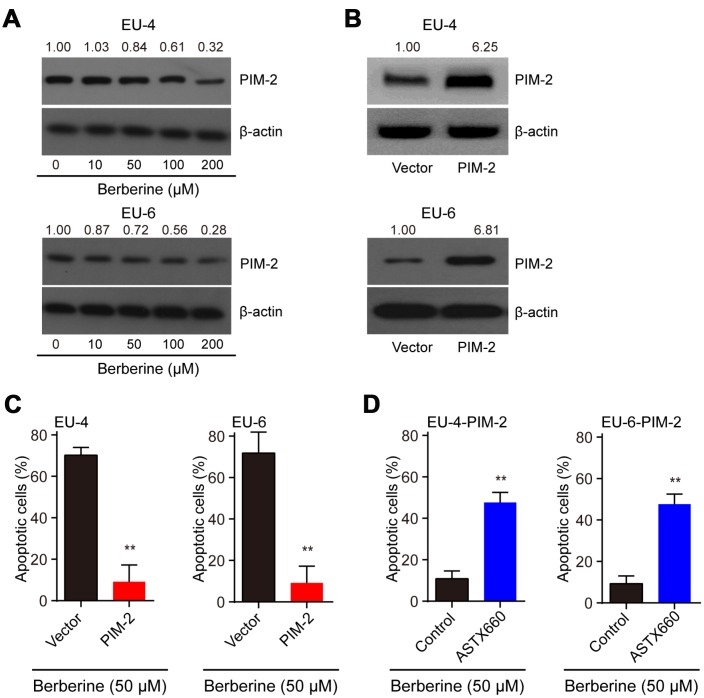
**BBR induces XIAP-mediated apoptosis through PIM-2.** (**A**) Representative western blot analysis of PIM-2 expression in ALL cells EU-4 and EU-6 treated with different concentration of BBR. (**B**) PIM-overexpressed EU-4 and EU-6 cells were treated with 50 μM BBR for 24 h. The transfection efficiency was determined using western blot. (**C**) Cell apoptosis was measured by TUNEL assay. (**D**) PIM-2 overexpressed EU-4 and EU-6 cells were treated with 50 μM BBR in the presence or absence of XIAP antagonist ASTX660 for 24 h followed by treatment with 50 μM BBR for 24 h. Cell apoptosis was detected by TUNEL assay. Results are expressed as mean ± SD.**p<0.01, compared to the vector or control group.

It reported that PIM-2 cooperates with XIAP to mediate cancer cells apoptosis [14]. To investigate the mechanism of XIAP-mediated apoptosis, we analyzed the expression of PIM-2 and found that the level of PIM-2 protein was dose-dependently downregulated in BBR-treated EU-4 and EU-6 cells (Figure 3A). It has been shown that PIM-2 kinase expression is involved in the compensatory survival mechanism of ALL cells due to its anti-apoptotic role [13]. We further evaluated apoptosis induced by BBR in the PIM-2 overexpressed EU-4 and EU-6 cells. Upregulation of PIM-2 significantly decreased the sensitivity of EU-4 and EU-6 cells to BBR (Figure 3B, 3C). XIAP is a downstream factor of PIM-2, raising the possibility that PIM-2/XIAP axis plays a role in BBR-induced apoptosis of EU-4 and EU-6 cells [14]. The results show that inhibition of XIAP with antagonist ASTX660 obviously attenuated BBR-induced apoptosis in PIM-2 overexpressed EU-4 and EU-6 cells (Figure 3D).

### MiR-24-3p mediated control of PIM-2 expression via targeting to 3’-UTR of PIM-2

**Figure 4:**
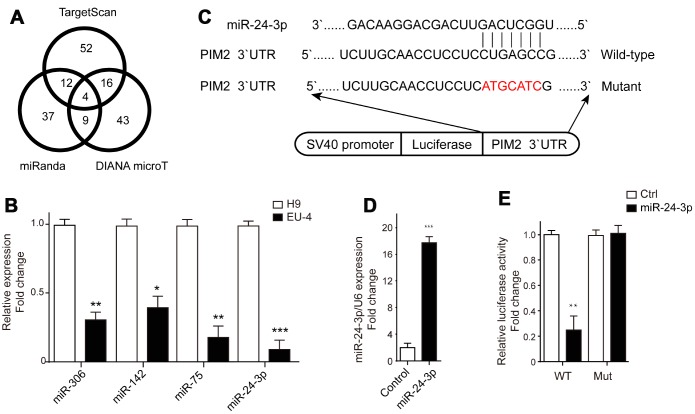
**PIM-2 is a target of miR-24-3p.** (**A**) The target microRNAs of PIM-2 were predicted by three prediction algorithms, TargetScan, miRanda and DIANA microT, and Venn diagram of overlap outcome between the prediction algorithms. (**B**) The expression profiles of microRNAs miR-306, miR-142, miR-75 and miR-24-3p in H9 and EU-4 cells. (**C**) The schematic diagram of the luciferase reporter gene plasmid containing the 3’-UTR of PIM-2 with predicted miR-24-3p target sites (Wild-type) or the mutated sites of 3’UTR of PIM-2 (Mutant). (**D**) The expression of miR-24-3p in HEK 293T cells transfected with miR-24-3p mimic (miR-24-3p) or negative control (Control). (**E**) The dual-luciferase reporter assay was performed in miR-24-3p-overexpressed HEK 293T cells. The numbers above the western blots band are the quantification of the gray value normalized to control group. Results are expressed as mean ± SD. *p<0.05, **p<0.01, ***p<0.001, compared to H9 or Control group.

The microRNAs targeting PIM-2 were predicted by three prediction algorithms, TargetScan, miRanda and DIANA microT. Four common microRNAs were identified, including miR-306, miR-142, miR-75, and miR-24-3p (Figure 4A). We examined the expression profiles of these four microRNAs in H9 and EU-4 cells and found that their relative expression in EU-4 cells was lower than that in H9 cells (Figure 4B). The luciferase reporter gene plasmids containing the 3’-UTR of PIM-2 with predicted miR-24-3p target sites (wild-type) or the mutated sites of 3’-UTR of PIM-2 (Mutant) were constructed (Figure 4C) and transfected into miR-24-3p-overexpressed EU-4 cells (Figure 4D). The dual-luciferase reporter assay confirmed that miR-24-3p obviously reduced the relative luciferase activity of luciferase reporter gene plasmids containing the 3’-UTR of PIM-2 with predicted miR-24-3p target sites, while few affected on the luciferase activity of luciferase reporter gene plasmids containing the mutated sites of 3’UTR of PIM-2 (Figure 4E).

### MiR-24-3p is implicated in the BBR-induced apoptosis by targeting PIM-2

**Figure 5:**
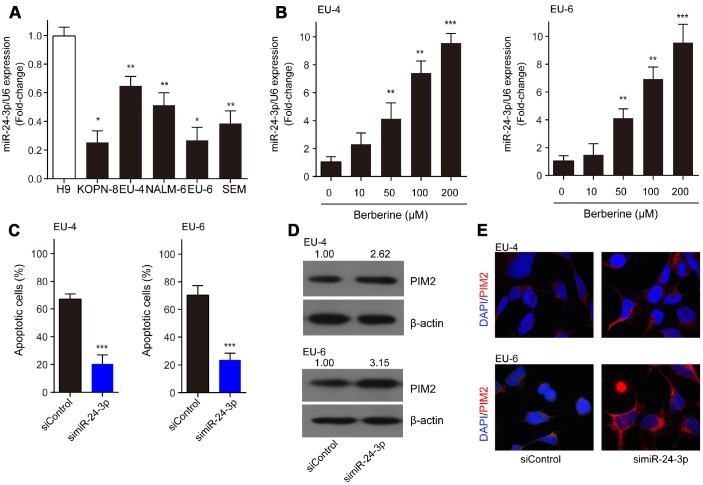
**MiR-24-3p is implicated in the BBR-induced apoptosis by targeting PIM-2.** (**A**) The expression profile of miR-24-3p in ALL cells, KOPN-8, EU-4, NALM-6, EU-6, SEM, and human lymphoma cell H9. (**B**) Effect of BBR on miR-24-3p expression in EU-4 and EU-6 cells. EU-4 and EU-6 cells were treated by BBR at the indicated concentrations for 24 h. The relative expression of miR-24-3p was measured by qRT-PCR and normalized to U6. EU-4 and EU-6 cells were transfected with siRNA targeting miR-24-3p (simiR-24-3p) or negative control (siControl), and subsequently treated with 50 μM BBR for 24 h. (**C**) Cell apoptosis was measured by TUNEL assay. (**D**) Representative western blot analysis showing PIM-2 expression in the cells. (**E**) Representative images of PIM-2 immunofluorescence staining in the treated cells. The numbers above the western blots band are the quantification of the gray value normalized to control group. Results are expressed as mean ± SD. *p<0.05, **p<0.01, ***p<0.001, compared to the indicated group.

Compared to H9 cells, miR-24-3p was significantly decreased in ALL cells, KOPN-8, EU-4, NALM-6, EU-6, and SEM (Figure 5A). Moreover, we detected increased expression of miR-24-3p in EU-4 and EU-6 cells following BBR exposure (Figure 5B). We investigated the role of miR-24-3p in the mechanism of BBR-induced EU-4 and EU-6 cells apoptosis. For this purpose, EU-4 and EU-6 cells transfected with siRNA targeting miR-24-3p (simiR-24-3p) or negative control (siControl) were treated with 50 μM BBR for 24 h. Inhibition of miR-24-3p rescued the BBR-induced apoptosis in EU-4 and EU-6 cells (Figure 5C). Further western blot and immunofluorescence assay revealed that inhibition of miR-24-3p increased the expression of PIM-2 protein in both EU-4 and EU-6 cells (Figure 5D, 5E).

### BBR extends the survival and attenuates the pathological conditions in ALL-transplanted xenograft mice

**Figure 6:**
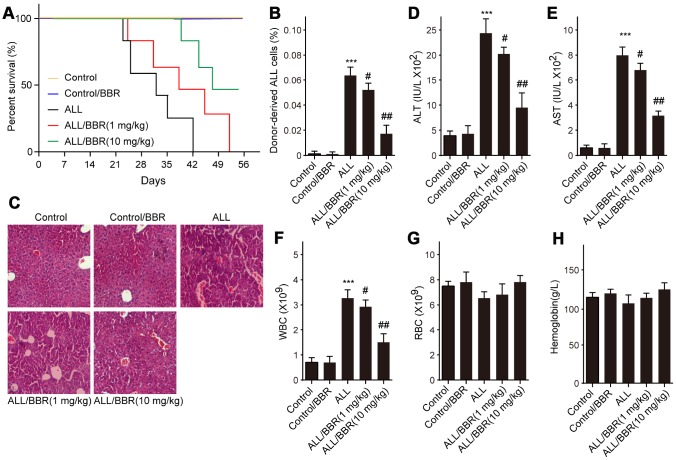
**BBR extends the survival and attenuates the pathological conditions in ALL-transplanted xenograft mice.** SCID mice, injected with either EU-4 cells or an equivalent volume of PBS via tail vein, were treated with BBR at 1 or 10 mg/kg (n=6 mice per group). (**A**) Kaplan-Meier survival curves showing the mortality of the mice. (**B**) Donor-derived ALL cells were analyzed by flow cytometry. (**C**) Representative images of H&E staining of liver tissues (magnification×). (**D**) The levels of alanine aminotransferase (ALT) and (**E**) aspartate aminotransferase (AST) in serum were measured. The number of (**F**) white blood cells (WBC) and (**G**) red blood cells (RBC) in blood. (**H**) Serum levels of hemoglobin. SCID mice, injected with wild-type (WT) or XIAP knockout EU-4 cells (XIAP^-/-^) via tail vein, were treated with 10 mg/kg BBR (n=6 mice per group). Results are expressed as mean ± SD. ***p<0.001, compared to the Control; #*p*<0.05, ##*p*<0.01, compared to ALL group.

Based on the above results, firstly, ALL-transplanted xenograft mice were established with EU-4 cells and administered with BBR at 1 or 10 mg/kg. Results showed that administration of BBR increased the survival rate of ALL-transplanted xenograft mice, as shown by the Kaplan-Meier curve (Figure 6A). In addition, the survival rate of ALL-transplanted xenograft mice in 10 mg/kg BBR group was higher than that in the mice with 1 mg/kg BBR (Figure 6A). Flow cytometric analysis showed that BBR at 10 mg/kg significantly decreased the number of donor cells in the mouse bone marrow (Figure 6A). Liver histology with H&E staining showed that BBR decreased ALL cells infiltration (Figure 6A). Moreover, BBR significantly reduced the serum levels of alanine aminotransferase (ALT) and aspartate aminotransferase (AST) in the mice compared to in the ALL group (Figure 6D and 6E). A significant increase in peripheral blood white blood cells (WBC) was observed in ALL-transplanted xenograft mice, but this increase was rescued by BBR administration (Figure 6F). There were no statistically significant differences in red blood cells (RBC) and hemoglobin between experimental group mice compared to control mice (Figure 6G and 6H).

### Inhibition of XIAP augments the effects of BBR on leukemia phenotype in xenograft mice

**Figure 7:**
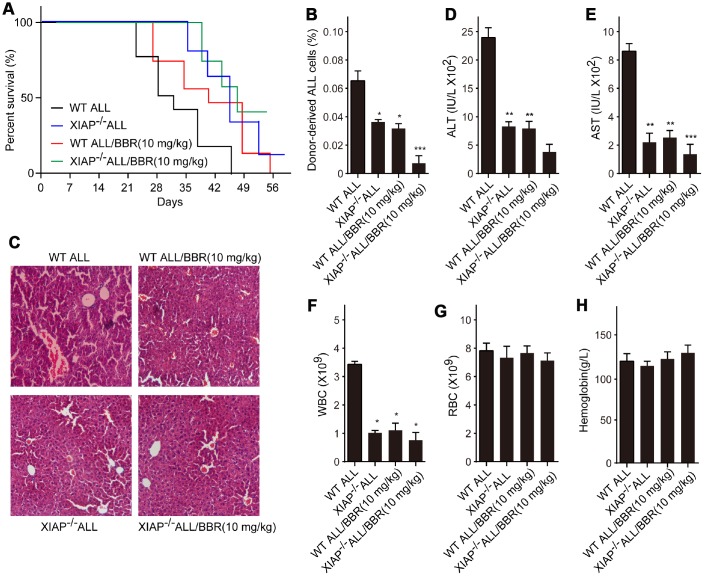
**Inhibition of XIAP augments the effects of BBR on leukemia phenotype in xenograft mice.** (**A**) Kaplan-Meier survival curves showing the mortality of the mice. (**B**) Donor-derived ALL cells were analyzed by flow cytometry. (**C**) Liver sections stained with H&E. (**D**) The levels of alanine aminotransferase (ALT) and (**E**) aspartate aminotransferase (AST) in serum were measured. The number of (**F**) white blood cells (WBC) and (**G**) red blood cells (RBC) in blood. (**H**) Serum levels of haemoglobin. Results are expressed as mean ± SD. *p<0.05, **p<0.01, ***p<0.001, compared to the Control or indicated group.

Subsequently, we established ALL-transplanted xenograft mice with XIAP knockout EU-4 cells (XIAP^-/-^), and the mice were treated with 10 mg/kg BBR. We found that XIAP knockout synergized with BBR treatment increased survival rate of ALL-transplanted xenograft mice (Figure 7A). XIAP knockout significantly decreased ALL cells infiltration in liver tissue and the number of donor cells in the mouse bone marrow as compared to BBR administration alone (Figure 7B and 7C). XIAP knockout also augmented the effects of BBR on the serum levels of ALT, AST and peripheral blood WBC (Figure 7D–7F). There were no statistically significant differences in RBC and hemoglobin of all group mice compared to the control mice (Figure 7G and 7H).

### Expression and correlations of XIAP, PIM-2 and miR-24-3p in ALL samples

**Figure 8:**
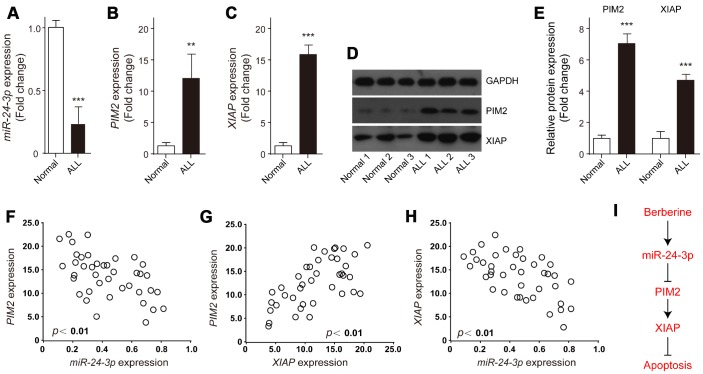
**Expression and correlations of XIAP, PIM-2 and miR-24-3p in ALL samples.** The relative expressions of (**A**) miR-24-3p, (**B**) PIM-2 and (**C**) XIAP in the bone marrow of ALL patients (n=39) or normal control (n=3) were detected by qRT-PCR. (**D**, **E**) Representative western blot and quantification analysis of PIM-2 and XIAP in ALL and normal samples. (**F**–**H**) Correlation analysis was performed between miR-24-3p, PIM-2 and XIAP in ALL patients. (**I**) The signaling pathway of BBR induced ALL cells apoptosis. Results are expressed as mean ± SD, **p<0.01, ***p<0.001, compared to Normal group.

The expression profiles of miR-24-3p, XIAP and PIM-2 in ALL samples were examined. The results showed that miR-24-3p was obviously decreased in ALL samples compared to the control (Figure 8A). In contrast, the relative expression levels of both XIAP and PIM-2 mRNA were significantly increased in ALL samples (Figure 8B and 8C), which were further validated by western blot analysis (Figure 8D, 8E). Of note, both PIM-2 and XIAP had obviously negative correlation with miR-24-3p in ALL samples (Figure 8F and 8H), whereas PIM-2 was significantly positively related to XIAP (Figure 8G). These data demonstrate that BBR induced ALL cells apoptosis via miR-24-3p-mediated PIM-2/XIAP signaling (Figure 8I).

## DISCUSSION

XIAP, also named as BIRC4, has been found to be ubiquitously expressed in multiple human cancer tissues and cell lines, and correlated with patients' poor prognosis [20]. XIAP plays an anti-apoptotic role by inhibition of caspases both in lymphoid and myeloid tumors [21]. Apoptosis is an important process to maintain homeostasis and morphogenesis in tissues. It is known that the expression of anti-apoptotic genes increases the risk of carcinogenesis. Resistance to apoptosis is also a pivotal reason for failure of chemotherapy and radiotherapy in patients with advanced cancer. Consequently, inhibiting XIAP has the potential to be a novel approach for cancer treatment. Some small molecules have been designed to restrain the expression of activity of XIAP, thereby promoting apoptosis progression, such as second mitochondrial activator of caspases (Smac) mimetics [22]. Small molecule inhibitors for XIAP reduce clonogenic survival and promote apoptosis in childhood ALL cells through inducing TRAIL-mediated activation of caspases [23]. BBR, a small molecule alkaloid purified from Chinese herbs, induces strong apoptosis in ALL cells that are positive for wild-type p53 [8]. Prior to this study, a report indicated that BBR induces p53-independent apoptosis in p53-null ALL cells by inhibiting XIAP [9]. In the present study, our data suggested that BBR significantly decreased the viability of p53-null leukemia cells EU-4 and p53-mutant leukemia cells EU-6, and induced cell apoptosis accompanied by increased cleavage of caspase-3. We suggest that BBR induced apoptosis of EU-4 and EU-6 cells, at least in part, by caspases activation, which was further confirmed by finding that broad-range caspase inhibitor z-VAD-FMK prevented BBR-induced apoptosis in both EU-4 and EU-6 cells. More importantly, results showed that BBR strongly inhibited XIAP expression, and demonstrated that XIAP was implicated in the BBR-induced apoptosis in EU-4 and EU-6 cells. High values of ALT, AST, and WBC are the significant predictors for ALL. In this study, we found that inhibition of XIAP augments the effects of BBR on leukemia phenotypes by suppressing the serum levels of ALT, AST and WBC in xenograft mice, indicating BBR alleviated ALL condition by suppressing XIAP.

Further investigating into the mechanism of action, we found that BBR also downregulated the level of PIM-2 protein in EU-4 and EU-6 cells. Human PIM-2 is a proto-oncogene involved in regulating cancer biology, including cell survival and tumorigenesis [24]. PIM-2 also negatively regulates anti-tumor response by restraining T cell responses [25]. It was found that PIM-2 contributes to the development of rapamycin resistance in hematopoietic cells [26]. The PIM kinase inhibitor, SGI-1776, has been shown to effectively induce apoptosis of chronic lymphocytic leukemia (CLL) cells and acute myeloid leukemia (AML) cells [27, 28]. In addition, PIM-2 kinase is involved in the compensatory survival mechanism of ALL cells through its anti-apoptotic role [13]. In this study, we showed that upregulation of PIM-2 significantly decreased the sensitivity of EU-4 and EU-6 cells to BBR, validating the important role of PIM-2 in the mechanism of BBR-induced apoptosis of p53-null and -mutant ALL cells. PIM-2 inhibited apoptosis of prostate cancer cells by downstream factor XIAP [14]. Similarly, we confirmed that BBR induced XIAP-mediated apoptosis through PIM-2.

Various studies have reported that BBR treatment could alter the expression of miRNAs, such as miR-23a [29], miR-101 [15], and miR-373 [30]. The potential mechanism of this regulation was still not entirely clear. Another important observation of the present study was that BBR primarily upregulated miR-24-3p, thus repressing its target PIM-2 expression. By using prediction algorithms and dual-luciferase reporter assay, we confirmed that PIM-2 was a target of miR-24-3p and miR-24-3p mediated control of PIM-2 expression via targeting to 3’-UTR of PIM-2. Many studies have suggested that miR-24-3p was closely related to human cancer progression, functioning as an oncogene or a tumor suppressor, such as in lacrimal adenoid cystic carcinoma, nasopharyngeal carcinoma, mesothelioma, and lung adenocarcinoma [31–34]. Compared to H9 human lymphoma cells, miR-24-3p was significantly decreased in ALL cells, KOPN-8, EU-4, NALM-6, EU-6, and SEM. And BBR upregulated miR-24-3p expression in EU-4 and EU-6 cells. Our data revealed that inhibition of miR-24-3p rescued the BBR-induced apoptosis in EU-4 and EU-6 cells and increased the expression of PIM-2 protein, as detected by western blot and immunofluorescence analysis.

Therefore, we provide evidence that miR-24-3p/PIM-2/XIAP axis played significant roles in the mechanism of BBR-induced apoptosis. A possibility exists that miR-24-3p, PIM-2, and XIAP may be dysregulated in ALL. As expected, miR-24-3p was obviously decreased in the ALL samples compared to the control, but both XIAP and PIM-2 were significantly increased in the ALL samples. Moreover, both PIM-2 and XIAP had obviously negative correlation with miR-24-3p in the ALL samples, while PIM-2 was significantly positively related to XIAP.

Our results indicate the important role of XIAP in BBR-induced apoptosis of p53-null and -mutant ALL cells and suggest potential underlying mechanism of its regulation. Namely, miR-24-3p induced by BBR targets PIM-2, which positively regulates XIAP, and subsequently promotes caspase-dependent apoptosis of ALL cells (Figure 8H). The data of this study explain the potential mechanism of BBR-induced apoptosis of p53-null and -mutant ALL cells.

## MATERIALS AND METHODS

### Patients and tissue samples

Bone marrow samples were obtained from 40 ALL patients, who were newly diagnosed between December 2015 and January 2018 at The First Affiliated Hospital of Zhengzhou University with informed consent according to Institutional guidelines. Another 3 samples from healthy donors served as control. The study was approved by the Ethics Committee of The First Affiliated Hospital of Zhengzhou University, and conducted according to the principles of the Declaration of Helsinki.

### Cell lines and cell culture

ALL cells, KOPN-8, EU-4, NALM-6, EU-6, SEM, human lymphoma cells H9, and HEK 293T cells were purchased from American Type Culture Collection (ATCC, Manassas, VA, USA). ALL cells and H9 cells were grown in RPMI-1640 media containing 10% fetal bovine serum (FBS, HyClone, Logan, UT, USA), 1% L-glutamine and 1% penicillin-streptomycin (Hyclone). HEK 293T cells were cultured in Dulbecco's modified Eagle's medium (DMEM) supplemented with 10% FBS and 1% penicillin-streptomycin. All cells were cultured in a humidified atmosphere at 37°C and 5% CO_2_. For BBR treatment experiments, cells were treated with different concentrations of BBR (1, 10, 50, 100 and 200 μM) for 24 h. For verifying assay, EU-4 and EU-6 were incubated 24 h with 50 μM BBR in the presence or absence of z-VAD-FMK (50 μM) followed by apoptosis detection.

### CCK-8 assay

The CCK-8 assay was performed to assess cell proliferation ability. Cells (2×10^3^ cells/well) were seeded into 96-well plates and incubated with BBR at the indicated concentrations for 24 h. After that 10 μl of CCK-8 reagent (Sigma, St. Louis, MO, USA) was added to each well for 2 h according to the manufacturer’s instructions. Cell viability was measured using a microplate reader (Bio-Rad, Hercules, CA, USA) at an absorbance of 450 nm.

### TUNEL assay

The terminal deoxynucleotidyl transferase-mediated X-dUTP nick end labeling (TUNEL) assay was used to assess cell apoptosis. The assay was performed with in situ cell death detection kit (Roche, Basel, Switzerland) according to the manufacturer’s instructions. For determination the activity of caspase-3, the caspase-3 Colorimetric Assay Kit (Abcam, Cambridge, UK) was used, and the experiments were conducted according to the manufacturer’s instruction.

### Western blot

Total protein was extracted from cell lines or human bone marrow samples using RIPA lysis buffer (Invitrogen, Carlsbad, CA, USA) according to the manufacturer’s instructions. Next, the protein concentration was detected using the BCA protein assay (Beyotime Biotechnology, Shanghai, China). Proteins were separated by 10% SDS-PAGE and transferred onto PVDF membranes. The membranes were incubated with 5% non-fat milk at room temperature for 2 h, and then incubated with rabbit anti-mouse primary antibodies, including anti-Pro-caspase-3 (ab32150, Abcam), -cleaved-caspase-3 (ab2302, Abcam), -XIAP (ab28151, Abcam), -PIM-2 (4730T, Cell Signaling) and -β-actin (3700S, Cell Signaling) at 4°C overnight. Then the membranes were incubated with secondary antibodies (Beyotime, Shanghai, China) at room temperature for 1 h. β-actin was used as an internal standard. The protein bands were visualized by electrochemiluminescence (ECL) assay (Millipore, Billerica, MA, USA).

### Qualitative Real-Time Polymerase Chain Reaction (qRT-PCR)

Total RNAs were extracted using TRIzol® reagent (Invitrogen, USA) and reverse transcribed into cDNA by PrimeScript RT reagent Kit (Takara). SYBR Green qPCR Master Mix (Takara) was used for qRT-PCR experiment. U6 or GAPDH was used as an internal standard.

### Cell transfection

For XIAP and PIM-2 overexpression, as well as miR-24-3p knockdown, XIAP and PIM-2 overexpressing plasmids or negative vector, and siRNA targeting miR-24-3p or their negative control were purchased from RiboBio (Guangzhou, China) and transfected into EU-4 and EU-6 cells by Lipofectamine 2000 (Invitrogen) according to the protocol of the manufacturer.

### Dual-luciferase reporter assay

The luciferase reporter gene plasmids (Promega Corporation, Madison, WI, USA) containing the 3’-UTR of PIM-2 with predicted miR-24-3p target sites (Wild-type) or the mutated sites of 3’UTR of PIM-2 (Mutant) were constructed. HEK 293T cells were then transiently co-transfected with the above luciferase reporter gene plasmids and miR-24-3p mimic (miR-24-3p) or negative control., Firefly and renilla luciferase activities were measured 48 h after co-transfection using a dual-luciferase reporter assay system (Promega Corporation).

### Immunofluorescence staining

For immunofluorescence analysis, cells were blocked with 10% bovine serum albumin (BSA) for 1 h, and then incubated with primary antibody against PIM-2 overnight at 4°C, followed by incubation with secondary antibodies (AlexaFluor 568-conjugated, 1:5000 dilution) at room temperature for 1 h. The nuclei were stained with 2-(4-amidinophenyl)-6-indolecarbamidine dihydrochloride (DAPI). Samples were washed after each step with PBS by three times. An Axio Observer Z1 microscope equipped with a Zeiss LSM 5 Live DuoScan System under an oil-immersion ×40 objective lens (Carl Zeiss, Oberkochen, Germany) was used for the fluorescence visualization. Images were acquired by using ZEN 2012 software.

### ALL xenograft mice

For animal experiment 1, 30 NOD-SCID mice (6-8-week-old) were randomly divided into five groups (six in each), and 18 of the mice were injected with EU-4 cells (5×10^6^ cells/mouse) via tail vein. Six days after injection, the transplanted mice were treated with BBR at 1 or 10 mg/kg. For animal experiment 2, 24 NOD-SCID mice were randomly divided into four groups (six in each). Mice were injected with Wild Type (WT) or XIAP knockout EU-4 cells (XIAP^-/-^) via tail vein and treated with 10 mg/kg BBR. Bone marrow samples were collected for detecting donor-derived ALL cells by flow cytometry with antibodies against E2A/PBX1 (BD Biosciences, Franklin Lakes, NJ, USA). The study was approved by the Ethics Committee of The First Affiliated Hospital of Zhengzhou University.

### Blood sample analysis

Blood was extracted from the abdominal aorta after hepatectomy. Alanine aminotransferase (ALT) and aspartate aminotransferase (AST) levels were determined with commercial assay kits (Nanjing Jianchen Bioengineering institute, Nanjing, China). White blood cell (WBC), red blood cell (RBC), and hemoglobin (HGB) were determined by a blood counter.

### Histological staining

The paraffin-embedded samples were dewaxed by baking and rehydrated with graded alcohols and washed in distilled water. The sections were then stained with hematoxylin and washed with running water. The sections were then washed in ammonium hydroxide solution. Each section was rinsed by using graded ethanol, Eosin (Sigma-Aldrich) solution was used counterstain the sections. After additional rinses in ethanol, the sections were dehydrated in graded alcohols, cleared in xylene, and cover-slipped.

### Statistical analysis

All data analysis was performed using GraphPad Prism version 6.0 (GraphPad Software, San Diego California, USA). Two-tailed Student's unpaired t-test was used for data analysis between two groups. One-way ANOVA followed by Tukey's post-hoc test was used for multiple comparisons. *p* < 0.05 was considered significant.
